# Experimental Determination of the Dielectric Constant of Micellar Hydration Layer via Localized Surface Plasmon Resonance of Gold Nanoparticles

**DOI:** 10.1140/epje/s10189-026-00558-y

**Published:** 2026-02-20

**Authors:** Elizete J. Patel, Julia L. M. Carneiro, Rozane F. Turchiello, Sergio L. Gómez

**Affiliations:** 1 Physics Department, Ponta Grossa State University, Av. General Carlos Cavalcanti, 4748, Ponta Grossa, 84030-900 PR Brazil; 2 Physics Department, Federal University of Technology of Paraná, R. Doutor Washington Subtil Chueire, 330, Ponta Grossa, 84017-220 PR Brazil

## Abstract

**Abstract:**

The localized surface plasmon resonance (LSPR) of plasmonic nanoparticles can be used both for measuring the dielectric constant in which they are dispersed and for determining changes in the structure of the medium around them. In this work, we explored the shift in the LSPR of gold nanoparticles (AuNPs), obtaining the out-of-plane dielectric constant of the hydration layer of nanoparticles dispersed in aqueous solutions of sodium dodecyl sulfate for concentrations below and above the critical micelle concentration. A reduction is observed, which is due to a soft confinement between the nanoparticle and the micelles. The confinement favors the in-plane alignment of the water’s molecular dipoles, hindering a rotation out-of-plane and reducing the tendency to align with an external electric field, i.e., diminishing the medium’s polarization.

**Graphical Abstract:**

The short penetration depth of the localized surface plasmon in a gold nanoparticle enables probing of the dielectric constant of a nanometer-scale layer near the interface, where water molecules are more highly organized than in bulk water. The presence of micelles further intensifies the structuring of water near the nanoparticle interface.
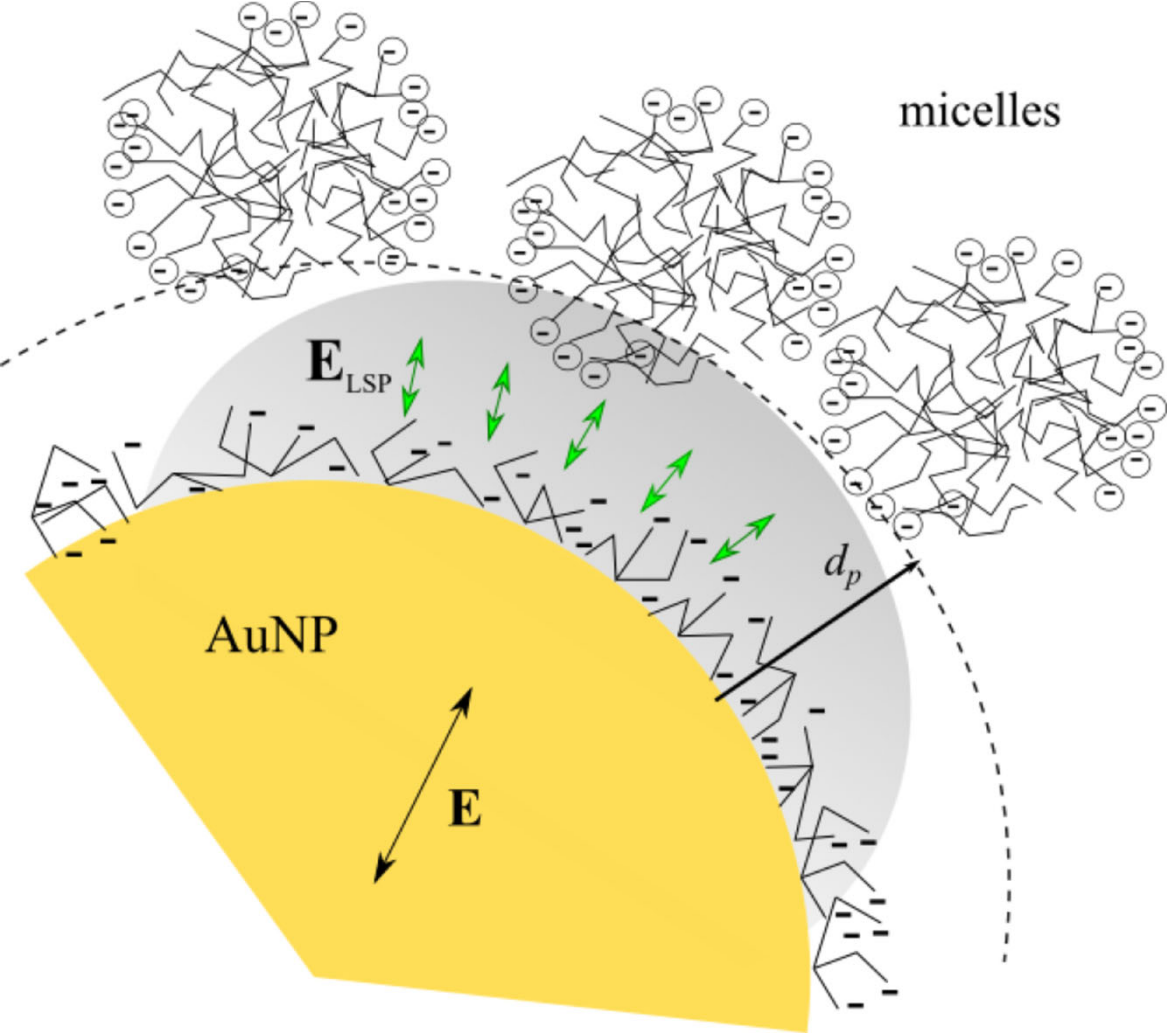

## Introduction

The dielectric constant or relative permittivity of a medium is a measure of the degree of polarization induced in it in the presence of an electric field, conditioning the interaction between charged species inside a dipolar liquid. Its value depends on the individual molecular response, measured by polarizability, and the collective response to an electric field. One particularly interesting case is that of interfacial water. Due to its influence on the strength of intermolecular forces, the dielectric constant within a nanometer-scale interfacial layer plays a crucial role in many processes, including ion solvation, surface hydration, molecular transport across membranes, and chemical reactions at electrodes [1, 2]. The tendency of a permanent molecular dipole to orient along the electric field direction to minimize energy, as in water, is reduced by interactions that decrease mobility. It is known that water in nanometer-scale geometries exhibits a decrease in its dielectric constant compared to its bulk value due to the reduction in mobility caused by confinement [3, 4]. Studies of strongly confined water have shown an almost vanishing dielectric constant for the out-of-plane component of the surface ($\varepsilon _{\perp }\sim$ 2) [2]. Previous work demonstrated the possibility of tuning both components of the dielectric constant by controlling the surface wettability [5]. On the other hand, the reduction is more pronounced for the dielectric constant in the direction perpendicular to the confining surface $\varepsilon _{\perp }$ compared to the parallel direction $\varepsilon _{\parallel }$. The dynamics of water molecules inside reverse micelles revealed the existence of different regimes as a function of micelle size, showing that the effect of the charged surface is short-ranged, along with a reduction in relaxation [6]. The decrease in orientational diffusion is attributed to the hydrogen bonding network of interfacial water [6, 7].

A standard method for determining the local dielectric constant employs molecular probes whose light absorption changes dramatically (solvatochromism) depending on the dielectric constant of the surrounding medium [8, 9]. Another type of probe for assessing the degree of polarization of a medium is plasmonic nanoparticles (NPs), such as noble metal nanoparticles of Ag and Au. Plasmonic nanoparticles have an absorption spectrum that presents a characteristic band due to the localized surface plasmon resonance (LSPR) in the visible range of the electromagnetic spectrum. The wavelength corresponding to the LSPR depends on factors such as the nanoparticle’s size and shape, and the medium’s dielectric constant surrounding the nanoparticle [10–12]. Under resonant excitation, the localized surface plasmon depends on the out-of-plane dielectric constant, due to the oscillating dipole of the plasmon. So plasmonic nanoparticles constitute an ideal label-free probe of the dielectric constant of a medium. Although silver and gold have similar electron densities, the intensity of the induced electromagnetic field at resonance on the surface of the nanoparticle is lower for gold than for silver. The difference arises because gold exhibits stronger plasmon dissipation, mainly due to a higher electron–phonon coupling—which results in shorter relaxation times [13]—and a lower interband transition energy, with its threshold lying in the visible range of the electromagnetic spectrum [14]. Consequently, the dipolar field of gold nanoparticles remains significant over shorter distances, i.e., the region probed by the plasmon of an Au nanoparticle is closer to the surface of the nanoparticle than in the case of an Ag nanoparticle. Previous works explored the utilization of the LSPR in Ag nanoparticles as a probe for determining the CMC of cationic surfactants [15–17]. Also, change in the maximum of the LSPR band of gold nanoparticles has been used to determine phase transition in lyotropic liquid crystals [18].

Exploiting the short penetration depth of the plasmon in gold nanoparticles, this work propose the employment of gold nanoparticles (AuNPs) as local probes for determining the effective out-of-plane dielectric constant of the medium around AuNPs, unveiling the changes in the structures of the hydration shell at the CMC of the sodium dodecyl sulfate (SDS)–water mixture.

**Fig. 1:**
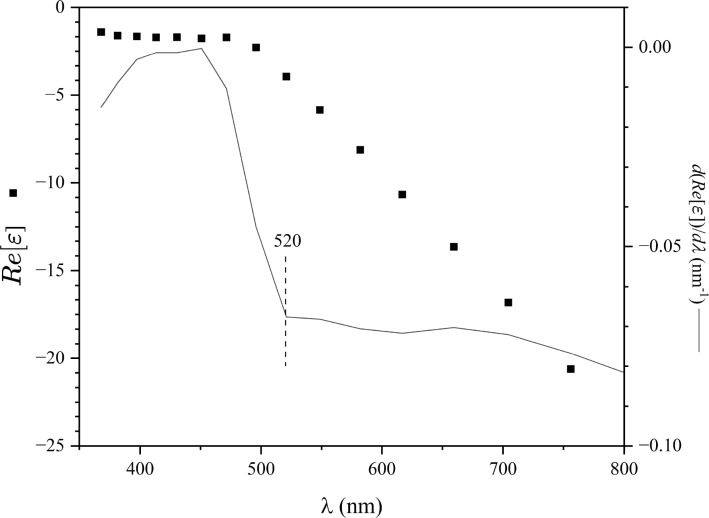
Graphs of the real part of the dielectric constant $\left( Re\left[ \varepsilon \right] \right)$ and its derivative $\left( {\text {dRe}}\left[ \varepsilon \right] /d\lambda \right)$ as a function of $\lambda$. Source ref. [14]

## Fundamentals

Coupling an electromagnetic field with a sub-wavelength metallic nanoparticle excites a non-propagating excitation termed localized surface plasmon. The nanoparticle’s surface furnishes a restoring force for the driven oscillating conduction electrons, giving rise to resonance. Following Maier’s formalism [10], the optical properties (absorption and scattering cross sections) of metallic nanoparticles of size *d* can be obtained within the framework of electromagnetism under the quasi-static approximation provided $d\ll \lambda$ [19]. In this way, the nanoparticle can be considered immersed in a homogeneous electric field. The polarizability $\alpha$ of spherical particles of radius *a* immersed in a medium of dielectric constant $\varepsilon _{\text {m}}$ under illumination with light of wavelength $\lambda$ is given by:

1 $$\begin{aligned} \alpha =4\pi a^{3}\frac{\varepsilon \left( \lambda \right) -\varepsilon _{\text {m}}}{\varepsilon \left( \lambda \right) +2\varepsilon _{\text {m}}}. \end{aligned}$$

A resonant condition is obtained by minimizing the modulus of the denominator. For a slowly varying condition of $\varepsilon$ around the resonance frequency, the minimum is reached if

2 $$\begin{aligned} \text {Re}\left[ \varepsilon \left( \lambda \right) \right] =-2\varepsilon _{\text {m}}, \end{aligned}$$

known as Fröhlich condition [20]. Consequently, a change in the dielectric constant in the medium will lead to a change in the wavelength at which resonance is observed. From the resonance condition (Eq. 2), a change $\Delta \varepsilon _{\text {m}}$ in the dielectric constant of the medium produces a change of the wavelength at the resonance ($\Delta \lambda _{\textrm{LSP}}$) given by:

3 $$\begin{aligned} \Delta \lambda _{\textrm{LSP}}=-2\left( \frac{{\text {dRe}}\left[ \varepsilon \right] }{d\lambda }\right) ^{-1}\Delta \varepsilon _{\text {m}}. \end{aligned}$$

The real part of the dielectric constant of a medium can be found from its refractive index (*n*) and extinction coefficient ($\kappa$) data as

4 $$\begin{aligned} \text {Re}\left[ \varepsilon \right] =\sqrt{n^2+\kappa ^2}. \end{aligned}$$

Fig. 1 shows data of the real part of the dielectric constant of gold in the visible–near-infrared region, calculated from data found in [21]. At $\lambda =520$ nm, the slope $\frac{{\text {dRe}}\left[ \varepsilon \right] }{d\lambda }$ is $\sim -0.0676\,$ $\hbox {nm}^{-1}$.

**Fig. 2:**
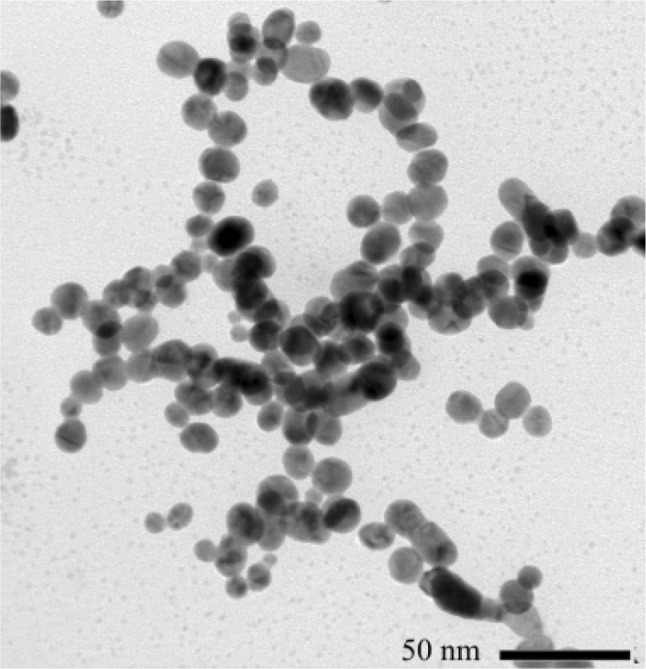
Image by TEM of the nanoparticles. Scale bar: 50nm

## Experimental details

### Synthesis of nanoparticles

**Fig. 3:**
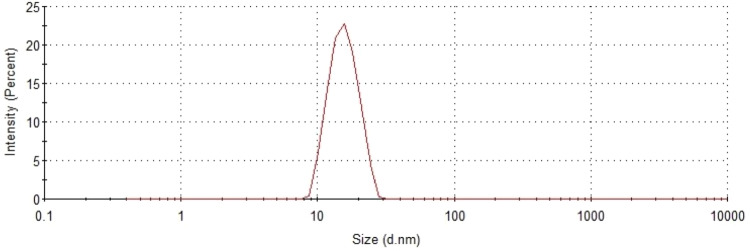
Size distribution intensity of the Au nanoparticles by DLS

The AuNPs were prepared by a photochemical route. Initially, 1.5 mL of a solution of 0.5 g of sodium citrate (Synth, ACS grade) in 50 mL of water was added to 15 mL of a 1 mM solution of gold (III) chloride trihydrate (Synth, ACS grade). All aqueous solutions were prepared using ultrapure water (Millipore Milli-Q^®^ Integral 5 Water Purification System). The as-prepared solution was illuminated with light of $\lambda =395$ nm for 60 min at room temperature, using commercial diodes with a nominal power of 50 W. The solutions were constantly agitated with a magnetic stirrer throughout the exposure [22]. The outcomes of the synthesis were characterized by transmission electron microscopy (TEM) (JEOL, JEM F200) and dynamic light scattering (DLS) (Zetasizer, Nano ZS90). The results are shown in Figs. 2 and 3, respectively. The TEM image reveals spherical nanoparticles with a uniform size distribution, which is corroborated by the DLS results.

For the aqueous solution of SDS (Synth), the volume of the colloid of AuNPs was considered in the water fraction. Conductivity measurements were taken with a conductivity meter DM-32 from DIGIMED, and UV–Vis spectra were collected with a Lambda 850+ from Perking Elmer which has a wavelength reproducibility $\le 0.020$ nm and an standard deviation of 10 measurements $\le 0.005$ nm. All the measurements were taken with a resolution of 0.5 nm.

## Results and discussion

**Fig. 4:**
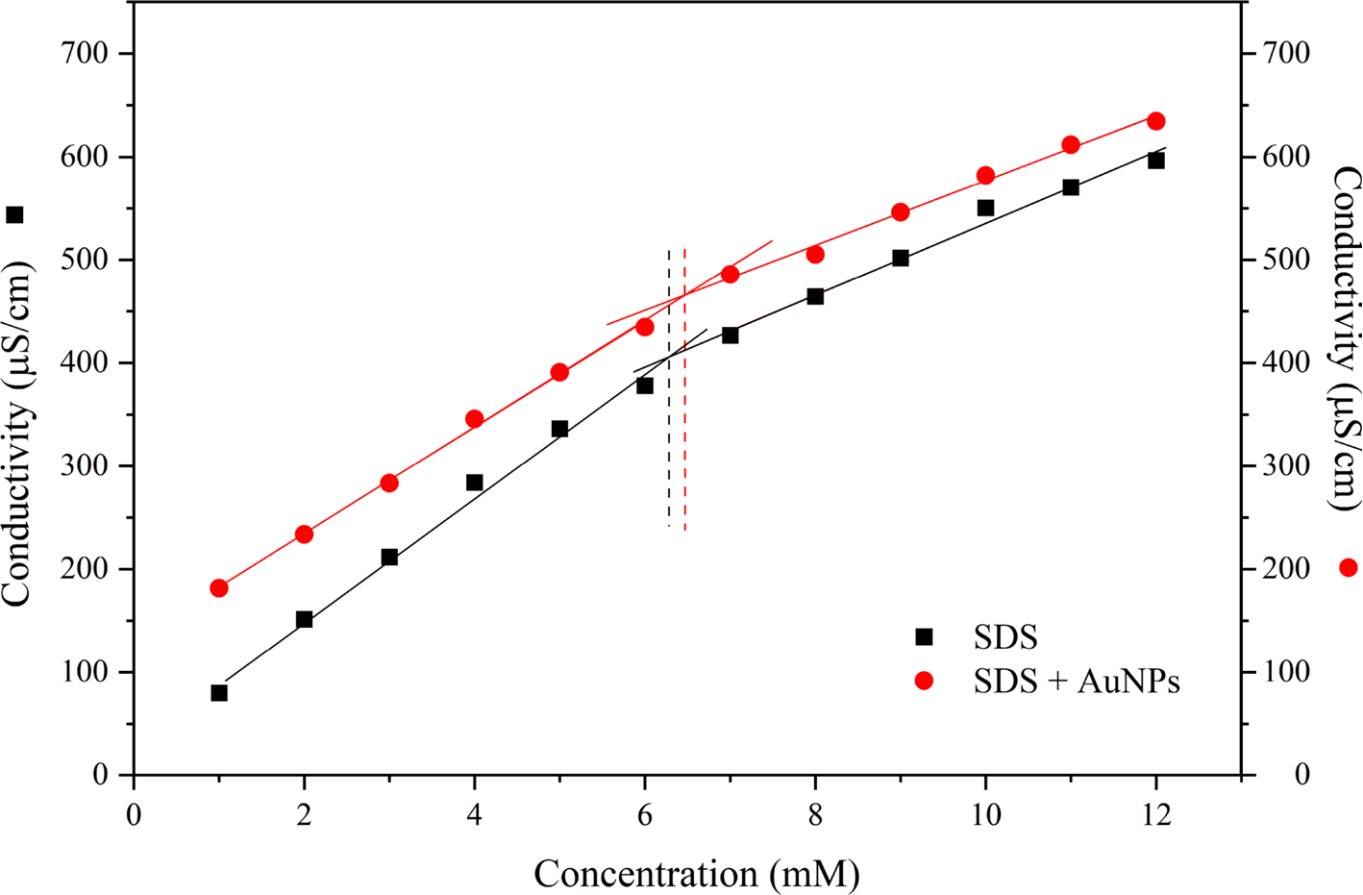
Conductivity of aqueous solutions of SDS, with and without AuNPs. Dotted lines indicate the CMCs of the samples

To determine the appropriate SDS concentration range for observing the CMC, we first measured the electrical conductivity of aqueous SDS solutions, both with and without AuNPs. The polar group of the amphiphilic molecule dissociates, releasing the counterion, which, in the case of SDS, is a sodium ion. The increase in amphiphilic molecules in the solution, and below the CMC, leads to an increase in the presence of ions and counterions in the solution, which leads to an increase in electrical conductivity. Above the CMC, the rate of increase in electrical conductivity with the increase in amphiphilic molecules decreases due to the formation of micelles. Due to the sulfate group, the micelles’ negative surface charge attracts sodium ions, leaving fewer charges available to contribute to electrical conductivity. Therefore, the angular coefficient of the electrical conductivity curve decreases above the CMC as a function of the number of amphiphilic molecules. The results are presented in Fig. 4. The values of the CMC were determined applying a segmented linear regression.

Figure 4 shows that adding AuNPs shifts the CMC from 6.88 to 6.45 mM, approximately, increasing the electrical conductivity’s value. Both values of CMC are in the known range for the CMC of SDS.

**Fig. 5:**
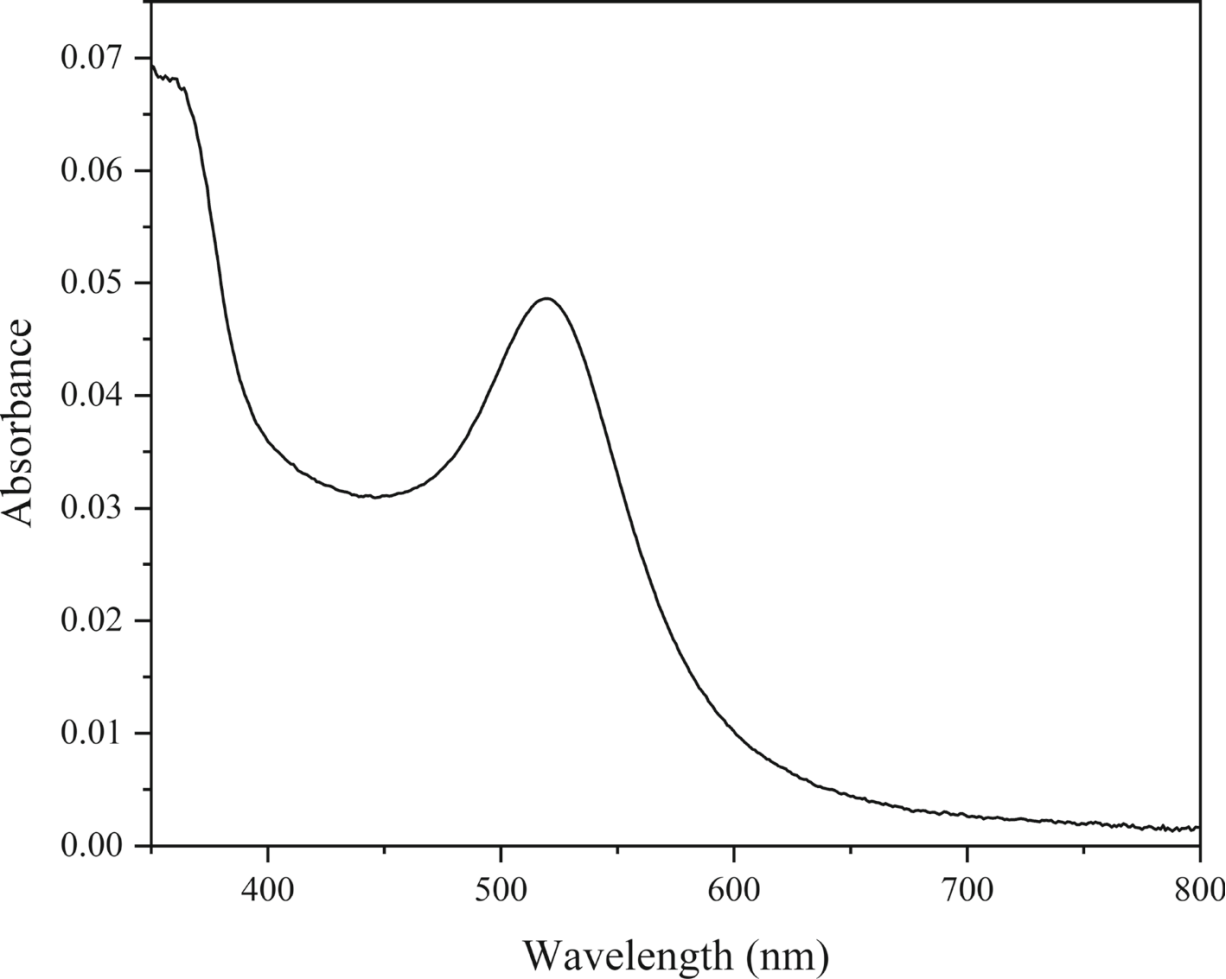
Typical LSPR band of the Au nanoparticles for a concentration of SDS below the CMC

Figure 5 shows a UV–Vis spectrum of aqueous solutions of SDS with AuNPs, exhibiting the typical LSPR band of AuNPs for a concentration below the CMC. From the Fröhlich condition, the value of the effective out-of-plane dielectric constant is $\varepsilon =1.946$. This value is rather higher than that of bulk water ($\sim 1.777$ at room temperature). These results can be understood due to the presence of the counter ions Na+ in the hydration layer of the nanoparticle. It is known that the addition of salt to otherwise pure water raises the value of the dielectric constant [23]. The results also showed no broadening of the band above the CMC due to any aggregation effect induced by micelle formation.

**Fig. 6:**
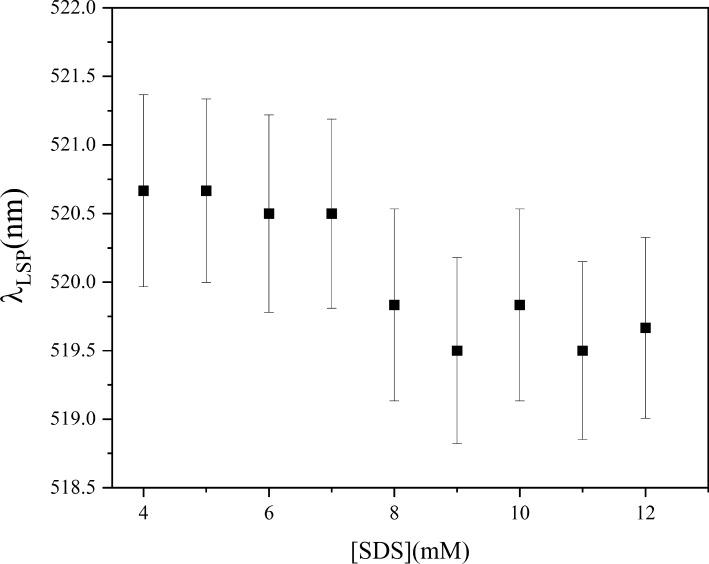
Wavelength at the maximum of LSPR peak ($\lambda _{\textrm{LSP}}$) as a function of SDS concentration

Figure 6 depicts the wavelength corresponding to the maximum of the LSPR as a function of the concentration of SDS. Each point is the average of three independent measurements..

**Fig. 7:**
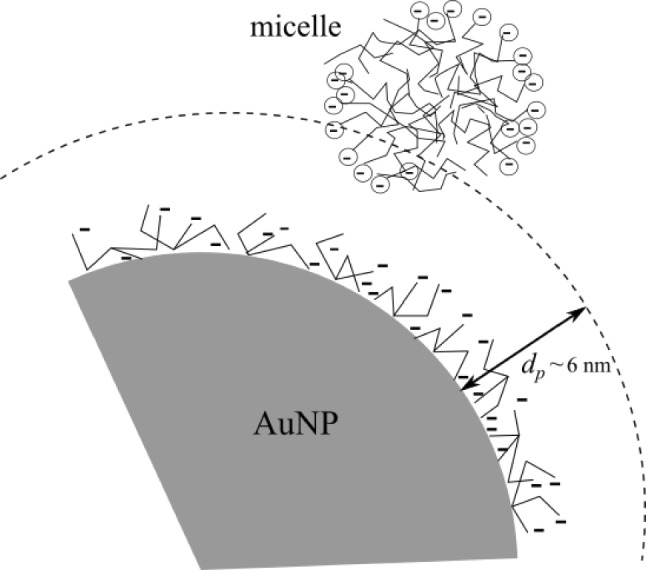
Schematic illustration of the spatial configuration between a gold nanoparticle and an SDS micelle. The interfacial region, corresponding to the confined hydration layer, is predominantly composed of structured water molecules with restricted rotational and translational mobility. This ordering alters the local dielectric environment, potentially influencing the plasmonic response of the nanoparticle

The value for the CMC of SDS obtained in this work agrees with the value reported by Salem et al., who employed the LSPR of AgNPs [16]. The shift of the CMC obtained via the LSPR to higher values was also observed by the same authors. Following (Eq. 3), the blueshift $\Delta \lambda _{\textrm{LSP}} = -1\,$nm corresponds to a change $\Delta \varepsilon =-0.0338$ of the dielectric function of the medium around the nanoparticle, i.e., the new value of the dielectric constant is $\varepsilon =1.9122$. This reduction of the dielectric constant of the medium can be attributed to a more ordered medium probed by the LSP. This value is in good agreement with that ones obtained experimentally [2] and by molecular dynamics (MD) simulations [3, 5, 24] of water under strong slab confinement. MD studies have also shown reduction of the dielectric constant in organic solvents [29]. The strength of the electric field around the plasmonic nanoparticle decays exponentially. The penetration depth $d_p$ is defined as the distance where the strength decays to 1/*e* of its maximum reached at the nanoparticle’s surface. For AuNPs of 20 and 40 nm, the $d_p$ is about 5.5 and 11.7 nm, respectively [25] (Fig. 7). On the other hand, water molecules around micelles exhibit a more ordered structure than bulk water. Numerous studies by molecular dynamics have allowed us to gain insight into the structure of the SDS micelle. A study conducted by Chun et al. [26] showed that the hydrophilic sulfate groups partially cover the surface of the micelle, meaning that the hydrophobic alkyl chains occupy a substantial portion of the surface. Both portions of the surface induce a reduction in the mobility of the water molecules. The motion of water dipoles in the two hydration layers near the headgroup oxygens, each typically hydrogen-bonded with five to six water molecules, is reduced by up to an order of magnitude compared to that in bulk water [27, 28]. In contrast, near extended hydrophobic surfaces, like the portion of the surface of the micelle occupied by the alkyl chains, water molecules are more ordered, forming up to three hydrogen bonds per molecule with other water molecules [1]. In this context, within a $\approx 6$-nm-thick interfacial layer surrounding the nanoparticle, water molecules exhibit a less isotropic arrangement than bulk water [28], resembling some aspects of ice’s crystalline structure, whose dielectric constant is lower than that of liquid water [24, 29], making plausible a lowering of the dielectric constant at the CMC.

Previous work employing AgNPs for probing CMC of SDS reported a redshift of the LSPR [16], corresponding to an increase of $\varepsilon _{\text {m}}$ above the CMC. It is known that the penetration depth of AgNPs is about 60 nm [30, 31], higher than for AuNPs [25], so a redshift is expected because the AuNP is probing mainly the core of the micelles, which is a rather homogeneous medium made of alkyl chains that have a higher dielectric constant than water.

## Conclusions

In conclusion, AuNPs proved to be sensitive and suitable probes of the local dielectric constant, demonstrating the detection of slight changes in the structure of the hydration layer due to the micellization of the amphiphiles. The results showed a diminution of about 0.0338 in the out-of-plane dielectric constant. This change can be attributed to the reduction of the diffusional rotation of the dipoles of water molecules in the layer of molecules localized between the surface of the nanoparticles and the micelles. This work highlights the possibility of using nanoparticles as local probes of the dielectric constant of a medium, particularly that of interfacial water. Potential applications of these nanoprobes include biology, for real-time monitoring molecular and cellular processes; chemistry, for studying catalytic and interfacial phenomena; and microfluidics, for monitoring processes in confined volumes. The possibility of synthesis with fine size control and their functionalization, further enhances their use for monitoring complex systems. One promising application is their use in near-field plasmonic optical microscopy, an adaptation of near-field scanning optical microscopy, using a single nanoparticle at the end of an optical fiber, acting as a nanoantenna.

## Data Availability

The data generated in this study are available from the corresponding authors upon reasonable request.
